# List of reviewers for *Renal Failure* 2021

**DOI:** 10.1080/0886022X.2021.2016226

**Published:** 2022-01-20

The Editors of *Renal Failure* would like to thank all of our reviewers for their contribution and support during 2021. High-quality peer review is essential to the success of the Journal and we greatly appreciate the dedication of all those who have contributed to this. 

A. Al-Amin, Maha

A. Kaysen, George

Abdul Salim, Sohail

Abraham, Georgi

Abreo, Ken

Abreu, Rui

Abud, Burçin

Achanti, Anan

Adedapo, Adeolu

Aeddula, Narothama Reddy

Afkari, Rouhi

Aftab, Raja Ahsan

Agar, Baris U.

Agostinho Cabrita, Ana de Lurdes

Ahn, Curie

Ahn, Sung Soo

Ai, Jun

Aigner, Christof

Akalin, Aysen

Akan, Mert

Akdam, Hakan

Akkoyun, Dursun

Akşit, Dilek

Akturk, Ibrahim Faruk

Akyuz, Aydin

Al Beladi, Fatima

Al Jurdi, Ayman

Al Musaimi, Othman

Al Shukaili, Ahmed

Al-Hwiesh, Abdulla K.

Al-Rawashdeh, Sami

Alam, Gulzar

Alfano, Gaetano

Ali, Ahlam

Ali, Hatem

Ali, Sameh

Allam, Sridhar

Almeida, Lucas Ferreira de

Alparslan, Caner

Alshogran, Osama

Amasyali, Akin Soner

Amirteimoury, Morteza

Ammar, Yaser

An, Won Suk

Andrade-Oliveira, Vinicius

Andronesi, Andreea

Angara, Sureshbabu

Angioi, Andrea

Annigeri, Rajeev

Ansari, Naheed

Antonic, Miha

Apostolou, Theophanis

Arany, Istvan

Ardalan, Mohammad R.

Arellano-Mendoza, Mónica Griselda

Arfian, Nur

Ari, Elif

Arslan, Mustafa

Arslan, Uğur

Arun, Oguzhan

Arya, Dharamvir Singh

Asano, Manabu

Asicioglu, Ebru

Askar, Tunay K.

Assadi, Farahnak

Assumpcao, Lia

Ataoglu, Esra

Aufhauser, David

Auyanet, Ingrid

Avci, Esin

Avramovski, Petar

Awobusuyi, Jacob

Ay, Arzu

Ayar, Yavuz

Ayaz, Gülsel

Aydede, Sema K

Aydogdu, Nurettin

Azer, Samy A.

Aziz, Fahad

Aziz, Manal

Azoubel, Luana

Azzi, Yorg Al

Bagchi, Soumita

Bagcı, Gokhan

Bai, Ming

Bai, Yongheng

Bakkaloglu, Sevcan

Balamuthusamy, Saravanan

Balgradean, Cristian

Banaei, Shokofeh

Bao, Hao

Bao, Hui

Baron, Byron

Barrera-Chimal, Jonatan

Barretti, Pasqual

Barril, Guillermina

Bartosova, Maria

Barutcu Atas, Dilek

Basher, A.

Basic-Jukic, Nikolina

Basit, Sulman

Basturk, Taner

Basu, Rajit K.

Battaglia, Yuri

Bayliss, George

Bayrakci, Nergiz

Beck, Laurence H.

Becs, Gergely

Behairy, Maha

Beilstein, Christian

Beken, Serdar

Bellafronte, Natália Tomborelli

Bellos, Ioannis

Besant, Gini

Besarab, Anatole

Bevc, Sebastjan

Bhalla, Anshul

Bhatt, Girish Chandra

Bhattacharjya, Shantanu

Bhowmik, Dipankar

Bianchi, Maria Eugenia Victoria

Bihari Bansal, Shyam

Bilgili, Beliz

Bingol Ozakpinar, Ozlem

Binnetoglu, Emine

Biston, Patrick

Bjorneklett, Rune

Blaszczyk, Jan

Bleyer, Anthony

Bob, Flaviu

Bojoc, Suzana

Borulu, Ferhat

Bossini, Nicola

Bostan Gayret, Özlem

Bottillo, Irene

Boudhabhay, Idris

Bove, Tiziana

Bozkurt, Yasar

Brand, Serge

Branislava, Ilinčić

Brodsky, Sergey V.

Bruno, Stefania

Budisavljevic, Milos

Bunout, Daniel

Burdmann, Emmanuel

Buturovic, Jadranka

Büyükcam, Fatih

Büyükkaragöz, Bahar

Cai, Guang-Yan

Cai, Guangyan

Cai, Hong

Cai, Jieru

Cai, Junjun

Cai, Kedan

Çakır, Murat

Camargo, Carlos

Can, Özgür

Canacankatan, Necmiye

Canaud, Bernard

Cander, Soner

Canetta, Pietro A. A.

Cano-Europa, Edgar

Canpolat, Nur

Canpolat, Ugur

Cantekin, Işın

Cao, Dongwei

Cao, Qinghua

Cao, XueSen

Capone, Domenico

Capusa, Cristina

Caravaca-Fontán, Fernando

Carlos, Iribarren

Carron, Pierre-Louis

Castaneda, Jorge

Castellano, Giuseppe

Catar, Rusan

Causland, Finnian R. Mc

Cernaro, Valeria

Cetin, Nuran

Chacko, Bobby

Chade, Alejandro

Chai, Chengzhi

Chan, Jenq-Shyong

Chang, Chia-Chu

Chang, Jer-Ming

Chang, Wei-Wei

Chang, Wenxiu

Chang, Yu-Tzu

Charitaki, Evangelia

Charoenngam, Nipith

Chávez-Iñiguez, Jonathan S.

Cheang, Anna

Chen, Aiqun

Chen, Chaojin

Chen, Cheng-Hsu

Chen, Chien-Liang

Chen, Chunbo

Chen, Fei-Fei

Chen, Guochun

Chen, Hui

Chen, Jin-Bor

Chen, Jing

Chen, Juan

Chen, Jun-Feng

Chen, Kuan-Hsing

Chen, Kuan-Hsing

Chen, Lan

Chen, Lizhang

Chen, Min

Chen, Szu-Chia

Chen, Tung-Sheng

Chen, Wei

Chen, Xiang-Mei

Chen, Xiaonong

Chen, Xiongwen

Chen, Yue

Chen, Zhenwei

Chen, Zijin

Cheng, Bihuan

Cheungpasitporn, Wisit

Chew, Sophia

Chi, Xinjin

Chiang, Heng-Pin

Chien, Ching-Wen

Cho, Heeyeon

Cho, Kyu Hyang

Cho, Min Hyun

Choi, Eunhee

Choi, Hye Min

Chou, Chu-Lin

Chow, Kai Ming

Chuan, Junlan

Chudek, Jerzy

Chung, Sungjin

Chung, Sungjin

Ciavatta, Dominic J

Cicek, Omer Faruk

Cil, Onur

Çilingir, Özlem Tuğçe

Cirrik, Selma

Coca, Steven G.

Colbert, Gates

Colussi, Giacomo

Cormican, Sarah

Coronado, Jorge

Costa, José Abrão

Cseprekál, Orsolya

Cuevas, Santiago

Cui, Fangqiang

Cui, Liyan

Cui, Tianlei

Cui, Wenpeng

Cumhur Cure, Medine

Cüre, Erkan

Cyrino, Claudia

Dai, Chongshan

Dai, Houyong

Danser, A. H. Jan

Danta, Chhanda Charan

Dantas, Marcio

Daoud, Ahmed

Darouich, Sihem

De Francesco Daher, Elizabeth

de Menezes Montenegro, Fabio Luiz

de Moraes, Thyago Proenca

de Pont, Anne-Cornelie

Dede, Semiha

Deira, Javier

Delanaye, Pierre

Delsante, Marco

Demir, Erol

Demirci, Cenk

Deng, Jin

Deveci, Köksal

Dhanapriya, Jeyachandran

Di, Jia

Dianat, Mahin

Diao, Changhui

Diao, Zongli

Dias, Cristiane

Dias, Cristiane Bitencourt

Diena, Davide

Dimko, Mira

Dincel, Gungor

Ding, Jianxun

Ding, Wei

Ding, Xiaoqiang

Diskin, Charles

Dixit, Mehul

Dixit, Shilpi

Djukanovic, Ljubica

Dobrovolskaia, Marina A.

Dogan, Ibrahim

Dogra, PavitraManu

Dokur, Mehmet

Domagala, Piotr

Dominijanni, Sara

Dong, Guiying

Donmez, Osman

Dos Santos, Daniela

Dreisbach, Albert

Du, Xin

Duan, Lian

Duan, Shao-Bin

Duan, Shao-Bin

Dudziak, Maria

Duława, Jan

Durmaz, Mehmet Sedat

Dursun, Murat

Dusso, Adriana S

Dwivedi, Amit

DyabAllawi, Ali

Dzekova-Vidimliski, Pavlina

Egrilmez, Mehtap

Eidi, Maryam

Eirini, Grapsa

Ekart, Robert

El Kassas, Mohamed

El Nakib, Gehad

El-Reshaid, Kamel

Elbarbary, Hany

ElKashab, Sahier

Elsheemy, Mohammed

Eraslan, Ersen

Eren, Hilal

Eren, Necmi

Eriguchi, Masahiro

Erkalma Şenateş, Banu

Erkut, Bilgehan

Erlandsen, E. J.

Eroglu, Murat

Ersoy, Fettah

Escalante, Bruno

Esen, Bennur

Eşkazan, Ahmet Emre

Esquivias, Elvira

Etezadi, Farhad

Etta, Praveen Kumar

Fabbian, Fabio

Fan, Jie

Fan, Junming

Fang, Wei

Fang, Yu-Wei

Fanni, Daniela

Faria, B.

Fayed, Ahmed

Fayh, Ana Paula

Feng, Sheng

Feng, Zhe

Fenoglio, Roberta

Feriozzi, Sandro

Ferraioli, Giovanna

Ferreira, Viviane

Fiedler, Roman

Figueiredo, Ana Carolina

Figurek, Andreja

Fila, Branko

Filiopoulos, Vassilis

Fiorina, Paolo

Flannery, Alexander H.

Floccari, Fulvio

Fu, Jianhui

Fu, Ping

Fu, Xuebin

Fujisaki, Kiichiro

Fukasawa, Hirotaka

Fülöp, Tibor

Gadola, Liliana

Gadonski, Giovani

Gage, Shawn M

Gaipov, Abduzhappar

Gal-Oz, Amir

Gala, Agnieszka

Galichon, Pierre

Gallazzini, Morgan

Gan, Gin-Gin

Gan, Liangying

Gao, Kun

Gao, Xuxia

Gao, Yan

Garcia-Prieto, Ana

Garfinkle, Michael

Garneata, Liliana

Gauthier, Alexandre

Ge, Ning

Geng, Xiaodong

Georgiadis, George

Geurts, Aron M.

Gharaibeh, Kamel

Ghosh, Sanjib Kumar

Gil, Amnon

Girisgen, Ilknur

Glassock, Richard

Godhani, Umesh

Gois, Pedro

Gok, Mustafa

Gökçay Bek, Sibel

Goknar, Nilufer

Gomez, Gonzalo

Gomez, Hernando

Gong, Yong

Goodchild, Traci

Goplani, Kamal R

Goto, Shunsuke

Gou, Shen-Ju

Goudas, Pavlos

Gregg, Lucile

Grupper, Ayelet

Gu, Wanjie

Gu, Mingjia

Gubensek, Jakob

Gui, Ding-Kun

Gui, Yuan

Gul, Cuma

Gulcicek, Sibel

Gulel, Okan

Gumrukcuoglu, Hasan

Gunay, Emrah

Gunaydin, Zeki

Gunes, Sibel

Guo, Chun

Guo, Min

Guo, Yuanyuan

Gupta, Ankur

Gupta, Krishan Lal

Gupta, Jeena

Gurler, Mujgan

Gyamlani, Geeta

H El-Kashef, Dalia

Hadžibegović, Irzal

Halwachs-Baumann, Gabriele

Hamad, Abdullah

Hamrahian, Mehrdad

Han, Mei

Hanset, Nicolas

Hao, Hua

Harada, Makoto

Haroon, Sabrina Wong Peixin

Hasbal, N. Baris

Hasimu, Buaijiaer

Hassan, Kamal

Hayashi, Terumasa

Hayat, Ashik

Hayiroglu, Mert Ilker

He, Fan

He, Juan

He, Lijie

He, Linhua

He, Peng

He, Qiqi

He, Xiaoshun

He, Ya-Ni

He, Yingdong

He, Zhangxiu

Heidari, Ghobad

Herberth, Johann

Hernández Garduño, Eduardo

Herzog, Rebecca

Heybeli, Cihan

Heyse, Alex

Hidaka, Sumi

Hiki, Masaru

Hiramitsu, Takahisa

Hiser, Wesley

Hogan, Susan

Holick, Michael F

Hong, Daqing

Hong, Quan

Hong, Yu Ah

Hongsakul, Keerati

Horinouchi, Yuya

Horvatic, Ivica

Hoscan, Mustafa Burak

Hosojima, Michihiro

Hosseinpanah, Farhad

Hosseinzadeh-Attar, Mohammad Javad

Hou, Weiping

Hsiao, Ching-Chung

Hsieh, Chun-Yih

Hsu, Bang-Gee

Hsu, Cheng-Kai

Hsu, Heng Jung

Hu, Zhigang

Hu, Bo

Hu, Henglong

Hu, Jie

Hu, Kebin

Hu, Shuai

Hu, Wenxue

Hu, Zhangxue

Huang, Xianghua

Huang, Biao

Huang, Chien-Wei

Huang, Gang

Huang, Hongdong

Huang, Kun

Huang, Qipeng

Huang, Wei-Lieh

Hung, Ming-Jui

Huo, Tehli

Hur, Mina

Ibarra, Cristina

Iguchi, Naoya

Ikeda, Yasumasa

Ikee, Ryota

Ikegaya, Naoki

Ikezumi, Yohei

Ilatovskaya, Daria

Ilgen, Ufuk

Imai, Naohiko

Inaguma, Daijo

Inal, Volkan

Indridason, Olafur S.

Inoue, Kosuke

Inston, Nicholas

Io, Hiroaki

Ishida, Akio

Ishii, Hideki

Ishiyama, Katsuya

Ishizu, Akihiro

Isidori, Andrea M.

Isitmangil, Gulbu

Islam, Mahmud

Ismail, Ammar

Ismail, Gener

Ito, Takashi

Iwasaki, Yoshiko

Iwazu, Yoshitaka

J, Manjunath

Jairam, Anantharam

Jalandhara, Nishant

Jamale, Tukaram

Jeloka, Tarun

Jemcov, Tamara K.

Jeng, Yachung

Jha, Vivekanand

Jia, Linpei

Jiang, Fen

Jiang, Hong

Jiang, Jun

Jiang, X. -Y.

Jiang, Yanfang

Jiang, Yuteng

Jin, Jianliang

Joensuu, Heikki

Jong, Gwo-Ping

Jorgetti, Vanda

Joshi, Kusum

Jouret, Francois

Julian, Bruce A.

Junglee, Naushad

Justa, Shivali

Justo, Dan

Kadihasanoglu, Mustafa

Kaewput, Wisit

Kafeshani, Marzieh

Kahraman, Dogan

Kai, Hirofumi

Kajbafzadeh, Abdol Mohammad

Kakajiwala, Aadil

Kamei, Daigo

Kamińska, Dorota

Kandir, Sinan

Kaneko, Shohei

Kang, Kyung Pyo

Kang, Seok Hui

Kaplan, Halil Mahir

Kaplan, Joshua

Karaboyas, Angelo

Karabulut, Ahmet

Karakala, Nithin

Karan, Mehmet Akif

Karataş, Mehmet Baran

Karimzadeh, Iman

Kasapoglu, Benan

Kaskel, Frederick

Kastritis, Efstathios

Kataria, Ashish

Katavetin, Pisut

Kato, Mitsuo

Kawano, Mitsuhiro

Kaya, Ahmet

Kaya, Yasemin

Kaygin, Mehmet Ali

Kayhart, Bryce

Kaynar, Mehmet

Kaypakli, Onur

Kazancioglu, Rumeyza

Ke, Lu

Kebapci, Nur

Kei, Anastazia

Keles, Nursen

Khalaf, Mohamed

Khan, Irfanullah

Khoury, Johad

Kiessling, Arndt-Holger

Kilis-Pstrusinska, Katarzyna

Kim, Hyo Jin

Kim, Gheun-Ho

Kim, Ki-Young

Kim, Su Hyun

Kim, Won

Kim, Yun-Bae

Kinashi, Hiroshi

Kirsch, Alexander H.

Kitamura, Mineaki

Kıvrak, Elfide Gizem

Klisic, Aleksandra

Klopotowski, Mariusz

Koch, Christian

Kocyigit, Ismail

Kohli, Harbir

Komaba, Hirotaka

Komaru, Yohei

Komatsuda, Atsushi

Kono, Keiji

Kopitko, Csaba

Kopp, Jeffrey B.

Korkmaz, Hasan

Koser, Murat

Kosugi, Takaaki

Kotani, Kazuhiko

Koukoulaki, Maria

Kovvuru, Karthik

Koyama, Teruhide

Krajewska, Magdalena

Kreepala, Chatchai

Krieter, Detlef H.

Kritmetapak, Kittrawee

Kulkarni, Yogesh A

Kuma, Akihiro

Kumar, Jitendra

Kumthekar, Anand

Kung, Vanderlene

Kuo, Ko-Lin

Kuriyama, Satoru

Kurtul, Alparslan

Kusztal, Mariusz

Kwon, Osun

Kwon, Soon Kil

Kwon, Young Joo

L'Imperio, Vincenzo

Lai, Carlo

Lai, Silvia

Lai, Tai-Shuan

lan, Ping

Langford, Marlyn

Larsson, Anders

Lau, Wei Ling

Laucyte-Cibulskiene, Agne

Lazaridis, Miltos

Le, Wei-Bo

Leblebicioglu, Hakan

Lee, Cheng-Chia

Lee, Chien-Te

Lee, Kuo-Hua

Lee, Su Mi

Leeaphorn, Napat

Lengvárszky, Zsolt

Lerant, Anna

Lerma, Claudia

Letachowicz, Krzysztof

Leung, Nelson

Lezaic, Visnja

Li, Can

Li, Chao

LI, Guisen

Li, Guoliang

Li, Huihui

Li, Jianying

Li, Jianzhong

Li, Kailin

Li, Longkai

Li, Mingcheng

Li, Peng

Li, Ping

Li, S.

Li, Shu

Li, Weizu

Li, Wenxiong

Li, Xiaolei

Li, Xiaoran

Li, Xiaozhong

Li, Xinli

Li, Xudong

Li, Yi

Li, Ying

Li, Yong

LI, Yuehong

Li, Zhanzhan

Liang, Kelly V.

Liang, Xinling

Liang, Xiubin

Liao, Xiaohui

Liern, Miguel

Lim, Soo Kun

Lin, Gen-Min

Lin, Hongli

Lin, Mercury

Lin, Tao

Lin, Yen-Chung

Lin, Yi-Ting

Lingaraju, Madhu C.

Lionaki, Sophia

Liou, Hung-Hsiang

Liu, Chung-Te

Liu, Shuangxin

Liu, Chan-Min

Liu, Giu-Ling

Liu, Hong

Liu, Juan

Liu, Jue

Liu, Longshan

Liu, Na

Liu, Qi-feng

Liu, Tongqiang

Liu, Wenhu

Liu, Xun

Liu, Xusheng

Liu, Yingjie

Liu, Yun

Liu, Yuyuan

Liu, Zhiwen

Liu, Zhongyan

Lo, Ying-Chih

Locatelli, Francesco

Long, Haibo

Lu, Chien-Lin

Lu, Fangping

Lu, Guoyuan

Lu, Kuo-Cheng

Lu, Renhua

Lu, Xiaohan

Lu, Ying

Lubas, Arkadiusz

Lucca, Leandro

Lukrafka, Janice

Lun, Hengzhong

Luo, Ping

Luo, Yang

Lydia, Aida

Schetz, Marie

Ma, Guang

Ma, Haoyang

Ma, Xiaofen

Ma, Yuan

Mahajan, Sandeep

Malhotra, Sheetal

Malik, Paul

Mallamaci, Francesca

Mallett, Andrew

Mallios, Alexandros

Malyszko, Jolanta

Mamenko, Mykola

Mansur, Juliana

Markić, Dean

Markóth, Csilla

Marques, Maria

Marques, Igor

Marti, Hans-Peter

Martin, William P.

Martino, Francesca

Martorana, Davide

Maruniak-Chudek, Iwona

Maruyama, Yukio

Masakane, Ikuto

Mathew, Roy O.

Matsumoto, Kotaro

Matsumoto, Yoshihiro

Matsuura, Ryo

Mazanowska, Oktawia

Mazhar, Kehkashan

Meier, Clemens M.

Melo, Zesergio

Mendonca, Satish

Meng, Juan

Meola, Mario

Mercadal, Lucile

Merlino, Giovanni

Metzinger, Laurent

Miao, Liying

Michael, Ashour

Mignani, Renzo

Miller, Jennifer

Milosevic, Danko

Mirzaei, Sepideh

Misof, Barbara M

Misra, Sanjay

Mitsopoulos, Efstathios

Miyazaki, Mariko

Mizerska-Wasiak, Magorzata

Mizota, Toshiyuki

Mizuno, Masashi

Mohebbati, Reza

Moktefi, Anissa

Moldovan, Diana

Molina-Jijon, Eduardo

Molnar, Miklos Zsolt

Molsted, Stig

Montgomery, Leslie David

Moosavi, Seyed

Morales-Buenrostro, Luis E.

Morishita, Yoshiyuki

Morito, Naoki

Morrone, Luigi

Mou, Shan

Mouspour, A.

Mubarak, Muhammed

Muciño Bermejo, María Jimena

Munoz-Felix, Jose M.

Musa, Noha

Mushahar, Lily

Musial, Kinga

Mušinović, Izeta Aganović

Myakala, Komuraiah

Myserlis, Paul

Na, Nin

Nada, Ritambhra

Nagahama, Kiyotaka

Nair, Sreeja

Naito, Yoshiro

Nakagawa, Naoki

Nakanishi, Koichi

Nakashima, Akio

Nakata, Junichiro

Namdar, Hossein

Nanayakkara, Nishantha

Naqvi, Rubina

Nardelli, Luca

Nasif, Wesam

Navar, L. Gabriel

Nayman, Alaaddin

Ndlovu, Kwazi C. Z.

Neciolu Orken, Dilek

Neugarten, Joel

Neves, Precil

Ng, Jack KC

Nghia, Vu

Nie, YuXin

Nigam, Lovelesh

Nigwekar, Sagar

Nikvarz, Naemeh

Ning, Yichun

Nisari, Mehtap

Nishimoto, Masatoshi

Nishino, Tomoya

Nochaiwong, Surapon

Noiri, Eisei

Nongnuch, Arkom

Nowicki, Michał

Nurmohamed, Shaikh

Oami, Takehiko

Obialo, Chamberlain

Obrisca, B.

Odiatis, Christoforos

Ogata, Hiroaki

Oh, Dong-Jin

Ohya, Masaki

Okamoto, Takayuki

Okamoto, Teppei

Oliveira, E. A.

Omboni, Stefano

Oneib, Bouchra

Orisakwe, Orish Ebere

Ossareh, Shahrzad

Ou, Santao

Ouyang, Chun

Oygar, Duriye

Ozaki, Iwata

Ozelsancak, Ruya

Ozen, Nurten

Ozkan, Gulsum

Ozturk, Savas

P S, Priyamvada

P. Szabó, Réka

Pajek, Jernej

Pakmanesh, Hamid

Pan, Min

Panagiotou, Angeliki

Pandak, Nenad

Pandya, Vaidehi

Papathanakos, Georgios

Pape, Lars

Parlakpinar, Hakan

Parv, Florina

Patel, Nilang

Pathak, Devendra

Pavlovic, Drasko

Pawlowicz, Ewa

Pawluczyk, Izabella

Paydas, Saime

Pedraza-Chaverri, José

Pedroso, Jose

Pehlivan, Sacida

Pei, Juan

Pembegul Yigit, Irem

Peng, Ai

Peng, Fenfen

Peng, Linlin

Peng, Xiaoli

Peng, Yu-Sen

Perazella, Mark

Pereira, Luciano

Perez Morales, Rosa Elena

Perna, Annalisa

Petejova, Nadezda

Pethő, Ákos

Petras, Dimitrios

Petrauskiene, Vaida

Petreski, Tadej

Petrova, Iliyana

Phelan, Paul

Phillips, Benedict L.

Pike, Mindy

Piko, Nejc

Pimentel, Ana

Pirozzi, Nicola

Pitt, Bertram

Pleniceanu, Oren

Podestà, Manuel

Poluan, Fellycia

Pontrelli, Paola

Prabhu, Ravindra

Praga, Manuel

Prakash Srivastava, Swayam

Provenzano, Michele

Provot, Francois

Puccini, Marco

Qi, Min-You

Qian, Jianchang

Qiao, Xi

Qin, Shu-Lan

Qin, Wei

Qiu, Ming-zhu

Quadri, Syed

Radanova, Maria

Radovic, Milan

Rai, Pradeep

Raimann, Jochen G.

Rajasekaran, Arun

Ralib, Azrina Md

Ramachandran, Raja

Ramratnam, Mohun

Randjelovic, Pavle

Rangan, Gopala

Rani Kanduri, Swetha

Raptis, Vassilios

Rathore, Hassaan A

Raucci, Angela

Ravn, Bo

Rázga, Zsolt

Ren, Hongqi

Ren, Huiwen

Ren, Wenkai

Rewa, O. G.

Roberti, Javier

Roberts Padde, John

Roberts, Matthew A.

Robertson, Francis P

Rocha, Paulo

Rodrigues, Juliana

Rodrigues, Vandilson Pinheiro

Rodrigues, Vinícius

Rodriguez, Mariano

Rohan, Vinayak

Rong, Ruiming

Roozbeh, Jamshid

Rosenberger, Christian

Rossi Feliciano, Marcus Antonio

Rotondi, Silverio

Rroji, Merita

Rubio, Jorge

Ruiyan, Wang

Russo, Elisa

Ruszkowski, Jakub

Ryffel, Bernhard

Saadat, Yalda

Sabath, Ernesto

Sabaz, Mehmet Süleyman

Sabry, Alaa

Sadeghnia, Hamid

Saedi, Babak

Sagan, Jiri

Sain, Milenka

Saito, Takao

Sakai, Ken

Sakai, Yukinao

Sakkas, Giorgos

Salim, Asmat

Samalavicius, Narimantas E.

Sancak, Eyup

Sanchez-Lozada, L. Gabriela

Sarioglu, Orkun

Sasaki, Takaya

Sato, Akinori

Sato, Eiichi

Satoh, Shigeru

Satoskar, Anjali A.

Sawanyawisuth, Kittisak

Sawires, Happy K.

Saydam, Onur

Sayin, Burak

Scarpioni, Roberto

Schneditz, Daniel

Schönmarck, Ulf

Seck, Sidy

See, Lai-Chu

Seeman, Tomas

Segelmark, Mårten

Seifi, Behjat

Seixas, Emerson

Selcuk, Nedim

Sequeira-Lopez, Maria Luisa S

Serafim, Clarita

Serafini, Gianluca

Sethi, Gautam

Sevillano, Angel M.

Seyahi, Nurhan

Seyler, Lucie

Sezer, Mehmet

Shacham, Yacov

Shang, Shun Lai

Shang, Wenjun

Shankarnarayanan, Divya

Shanmugam, Vijay

Shapiro, Joseph

Sharaf El Din, Usama

Sharma, Madhulika

Sharma, Sadhana

Sheikhbardsiri, Hojjat

Shen, Bo

Shen, Hong-chun

Shen, Huaying

Sheng, Feng

Shengyou, Yu

Shiao, Chih-Chung

Shigematsu, Takashi

Shinkov, Alexander

Shintaku, Sadanori

Shoorei, Hamed

Shum, Hoi Ping

Sikole, Aleksandar

Singer, Eric A.

Singh, Amrit Pal

Singh, Inderjeet

Singh, Jagmeet

Singh, Thakur Uttam

Singh, Tripat

Singh, Tripti

Siqueira, Flávia

Sja'bani, Mochammad

Śledziński, Tomasz

Snauwaert, Evelien

Soares, Viviane

Sofue, Tadashi

Soliman, Karim

Song, Nana

Song, Yu-Huan

Sousa, Clemente Neves

Soy, Ebru H. Ayvazoglu

Soysal, Pinar

Spoendlin, Julia

Srisawat, Nattachai

Srivali, Narat

Srivastava, Swayam Prakash

Stavroulopoulos, Aristeidis

Stefan, Gabriel

Steiger, Stefanie

Steinman, Theodore I.

Stepanova, Natalia

Stepien, Ewa

Sterner, Gunnar

Steubl, Dominik

Stibůrková, Blanka

Stoian, Dana

Stompór, Tomasz

Storr, Markus

Strohmaier, Walter Ludwig

Su, Baihai

Suchal, Kapil

Sugi, Motohiko

Sugimoto, Keisuke

Sugiyama, Noriyuki

Suleyman, Zeynep

Sullo, Nikol

Sumer, Ismail

Sun, Dong

Sun, Huili

Sun, In O.

Sun, Jing

Sun, Lin

Sun, Wei

Sun, Xinyuan

Sun, Xuefeng

Sung, Junne-Ming

Surmiak, Piotr

Susantitaphong, Paweena

Suzuki, Hitoshi

Suzuki, Yusuke

Sy, John

Szabó, Eva

Szabó, László

Taguchi, Takafumi

Taheri, Saeed

Taherkhani, Amir

Takahashi, Fumihiko

Takata, Tomoaki

Taliyan, Rajeev

Tamura, Kouichi

Tan, Ying

Tanabe, Katsuyuki

Tanaka, Hiroshi

Tanaka, Shigeru

Tanaka, Tetsuhiro

Tang, Patrick Ming

Tang, Rong

Tang, Wen

Tang, Ying

Tanimoto, Minako

Tao, Lijian

Tao, Xingjuan

Tapolyai, Mihály

Tasli, Funda

Teimouri, Amir

Teng, Xu

Tesar, Vladimir

Thajudeen, Bijin

Thamcharoen, Natanong

Thang, Le

Thibaudin, Damien

Thomas, Shannon D.

Thongprayoon, Charat

Tian, Jun-Ping

Tian, Lifang

Tiewsoh, Karalanglin

Tiong, Mark K.

Tisljar, Miroslav

Tokumoto, Masanori

Tokushige, Akihiro

Tombak, Anil

Tong, Fei

Tonyali, Senol

Tory, Kalmán

Toz, Huseyin

Trakarnvanich, Thananda

Tranche, Salvador

Trimarchi, Hernan

Trovato, Guglielmo

Tsai, Chang-Youh

Tsai, I-Jung

Tsai, Lung-Wen

Tsai, Shangfeng

Tsai, Yi-Chun

Tsalouchos, Aris

Tseke, Paraskevi

Tseng, Chin-Chung

Tsourous, George

Tsujita, Makoto

Tu, Hung-Pin

Tu, Xiao

Tunel, Huseyin Ali

Turgut, Faruk

Tziastoudi, Maria

Ubara, Yoshifumi

Uçar, Mehmet Ali

Ueno, Yuji

Ullah, Asad

Ullah, Kifayat

Ullian, Michael

Unis, Amina

Upadhyay, Ashish

Urushihara, Maki

Utariani, Arie

Uygun, Saime

Uzun, Mehmet

Vaingankar, Sucheta

Valeri, Anthony

Van Buren, Peter N.

Vangala, Chandan

Vanotti, Sandra

Varma, Ravi Prasad

Vega, Almudena

Velioglu, Arzu

Venneri, Mary Anna

Verbrugge, Frederik H.

Veres-Székely, Apor

Verma, Himanshu

VH, Chong

Vujičić, Božidar

Vujkovac, Bojan

Vychytil, Andreas

Wada, Jun

Wakino, Shu

Waks, Jonathan W.

Waly, Mostafa

Wan, Xin

Wan, Yigang

Wang, Fei

Wang, Changxi

Wang, Guyan

Wang, Hao

Wang, Hua

Wang, Huiming

Wang, Jiawu

Wang, Lihua

Wang, Ming-jie

Wang, Niansong

Wang, Ningning

Wang, Qiuyue

Wang, Su-Xia

Wang, Weiming

Wang, Xinchen

Wang, Yanhong

Wang, Zhilin

Wanjare, Shashir

Ward, Richard A.

Wei, Liang

Wei, Cheng-Yu

Weinhandl, Eric

Wen, Mei

Wesseling, Catharina

Widgren, Jennie Lönnbro

Wiecek, Andrzej

Wilson, Samuel Eric

Wolf, Jacqueline L

Wong, Jiunn

Wu, Chung-Kuan

Wu, Gang

Wu, Huijuan

Wu, Jianhua

Wu, Tai-Te

Wu, Xiaojing

Xia, Daozong

Xiao, Cheng-cheng

Xie, Xisheng

Xing, Li

Xiong, Jiachuan

Xiong, Mingxia

Xu, Dongmei

Xu, Feng

Xu, Guangtao

Xu, Guoshuang

Xu, Hong

Xu, Hui

Xu, Jiarui

Xu, Jiping

Xu, Nana

Xu, Tao

Xu, Tian

Xu, Yan

Xu, Yan

Xue, Cheng

Yadav, Raj

Yajima, Aiji

Yamada, Shunsuke

Yan, Jianyun

Yang, Chih-Yu

Yang, Chun

Yang, Fan

Yang, Guang

Yang, Hongtao

Yang, Li

Yang, Min

Yang, Qiongqiong

Yang, Won Seok

Yang, Xiao

Yang, Yanlang

Yang, Yingying

Yanlin, Huang

Yatim, Karim

Yazar, Hayrullah

Yazıcı Gencdal, Işıl

Ye, Hongjian

Ye, Bo

Ye, Guoxin

Yekta, Reyhaneh

Yeter, Hasan

Yi, Chunyan

Yi, Fan

Yi, Tiegang

Yildiz, Abdulmecit

Yilmaz, Akar

Yilmaz, Meral

Ying-Yong, Zhao

Yıldız, Fatih

Yılmaz, Mümtaz

Yokoyama, H

Yong-Gui, Wu

Yong, Tuck

Yoshida, Hidemi

Yoshida, Hisako

Yoshida, Tadashi

Yu, Chen

Yu, Elaine

Yu, Feng

Yu, Qing

Yu, Xiao-Yong

Yu, Xiaofang

Yu, Xiaoyang

Yu, Yuan

Yu, Zihua

Yuan, Qiongjing

Yuan, Xiao-Peng

Yuan, Yanggang

Yuan, Yujia

Yuksel, Selcuk

Zacharoulis, Dimitrios

Zahid, Salman

Zamani, Nasim

Zanetto, Alberto

Zanon, Jeferson

Zarbock, Alexander

Zayed, Bahaa

Zen, Ke

Zeng, Caihong

Zeng, Ming

Zeng, Rui

Zeng, Ying

Zhan, Xiaojiang

Zhang, Xiaoyan

Zhang, Yide

Zhang, Dongshan

Zhang, Dongliang

Zhang, Fan

Zhang, Guangyuan

Zhang, Haitao

Zhang, Hao

Zhang, Hong-Liang

Zhang, Jiandong

Zhang, Jin

Zhang, Kang

Zhang, Li

Zhang, Pan

Zhang, Qian

Zhang, Sen

Zhang, Suojian

Zhang, Wei

Zhang, Weiming

Zhang, Xiaojuan

Zhang, Yi

Zhang, Yu

Zhang, Zhongheng

Zhao, Fusheng

Zhao, Huiping

Zhao, Jinghong

Zhao, Li-Jun

Zhao, Man

Zhao, Min

Zhao, Ming-hui

Zhao, Shuangping

Zhe, Li

Zheng, Cai-Mei

Zheng, Chenfei

Zheng, Donghui

Zheng, Jin

Zheng, Ling

Zheng, Yi

Zhou, Chao

Zhou, Fei

Zhou, Fu-de

Zhou, Hua

Zhou, Li

Zhou, Lili

Zhou, Xiaoling

Zhou, Xiaoming

Zhou, Xiaoyan

Zhou, Yang

Zhou, Yilun

Zhu, Bin

Zhu, Jian-Hua

Zhu, Li

Zhu, Nan

Zhuo, Hui

Zhuo, Li

Ziamajidi, Nasrin

Zilberman-Itskovich, Shani

Zinkevich, Natalya

Zitt, Emanuel

Zoccali, Carmine

Zou, Jianzhou

Zou, Jianzhou

Zou, Peimei

Zou, Xiangyu

Zsom, Lajos

Zuo, Li

